# Machine Learning Model for Computer-Aided Depression Screening among Young Adults Using Wireless EEG Headset

**DOI:** 10.1155/2023/1701429

**Published:** 2023-05-31

**Authors:** Nazmus Sakib, Md Kafiul Islam, Tasnuva Faruk

**Affiliations:** ^1^Department of Electrical and Electronic Engineering, Independent University Bangladesh (IUB), Dhaka, Bangladesh; ^2^Biomedical Instrumentation and Signal Processing Lab (BISPL), Independent University Bangladesh (IUB), Dhaka, Bangladesh; ^3^Department of Public Health, Independent University Bangladesh (IUB), Dhaka, Bangladesh

## Abstract

Depression is a disorder that if not treated can hamper the quality of life. EEG has shown great promise in detecting depressed individuals from depression control individuals. It overcomes the limitations of traditional questionnaire-based methods. In this study, a machine learning-based method for detecting depression among young adults using EEG data recorded by the wireless headset is proposed. For this reason, EEG data has been recorded using an Emotiv Epoc+ headset. A total of 32 young adults participated and the PHQ9 screening tool was used to identify depressed participants. Features such as skewness, kurtosis, variance, Hjorth parameters, Shannon entropy, and Log energy entropy from 1 to 5 sec data filtered at different band frequencies were applied to KNN and SVM classifiers with different kernels. At AB band (8–30 Hz) frequency, 98.43 ± 0.15% accuracy was achieved by extracting Hjorth parameters, Shannon entropy, and Log energy entropy from 5 sec samples with a 5-fold CV using a KNN classifier. And with the same features and classifier overall accuracy = 98.10 ± 0.11, NPV = 0.977, precision = 0.984, sensitivity = 0.984, specificity = 0.976, and F1 score = 0.984 was achieved after splitting the data to 70/30 ratio for training and testing with 5-fold CV. From the findings, it can be concluded that EEG data from an Emotiv headset can be used to detect depression with the proposed method.

## 1. Introduction

Depressive disorder is a highly prevalent mental illness. Sadness, loss of interest or enjoyment, feelings of guilt or low self-worth, interrupted sleep or food, fatigue, and difficulty concentrating are some characteristics of depression. It may affect a person's capacity to operate in daily life or at work or school. According to the World Health Organization (WHO) back in 2015, almost 4.4% of the world's population was suffering from depression [1]. Because of the COVID-19 pandemic, many people suffered from depression due to job loss, study hampering, losing close relatives, staying indoors, etc. A study showed 19.3% increase in depression symptoms among people after COVID-19 in the United States [2]. A study has shown the changes in obsession, depression, and quality of life in schizophrenia patients before and after COVID-19 [3]. When depression is severe it can lead to suicide. Every year around 800 thousand people die because of suicide [1]. In 2017, 13.2% of young adults (aged 18–25) in the U.S. suffered from depression which was 5.1% less in the year 2009 [4]. Of the deaths of young people, around 9.1% are due to suicide [5]. In most suicide cases, people had psychiatric disorders where depression is the most common disorder among others [6]. According to a recent study, insecure attachment styles are linked to greater problems such as depression, social anxiety, and suicidal thoughts [7]. So, depression is a major issue that should be diagnosed and treated at an early age to prevent suicide and for the betterment of the quality of life.

There are various screening tools to detect depression. There are chronic social defeat stress models of depression such as the Morris water maze test and T-maze test to learn about the cognitive functions [8]. Traditionally, clinical questionnaire-based diagnoses are used to detect depression, where medical professionals (psychologists, psychiatrists, counselors, and physicians) interview and observe patients' behavior to determine depression [9]. The Multiple Sclerosis Depression Rating Scale (MSDRS) is a screening tool to evaluate depression in multiple sclerosis (MS) patients and may make fatigue and depressive symptoms more distinguishable [10]. Adjustment disorders with depressed mood are diagnosed using the Diagnostic and Statistic Manual of Mental Disorders, Fourth Edition, Text Revision (DSM-IV TR), published by the American Psychiatric Association (APA) [11]. The Beck Depression Inventory-II (BDI-II), a 21-item self-report questionnaire, is frequently used to assess the severity of depression in adults and adolescents. To be more congruent with DSM-IV depression criteria, the BDI-II was redesigned in 1996 [12]. The Center for Epidemiologic Studies Depression (CES-D) Scale, a commonly used self-report depression symptom scale, was given to convenience samples of high school and college students [13]. The most popular tool for patient selection and follow-up in research studies on depression treatments is the Hamilton Depression Rating Scale (HDRS, often known as HAM-D) [14]. The Depression Anxiety Stress Scales 21 (DASS-21) is reliable and can easily be used by the patient to understand their symptoms of depression, anxiety, and stress. It is based on 21 questionnaires, where 7 is for depression, 7 for anxiety, and 7 for stress [15]. The International Classification of Diseases 10th Revision (ICD-10) is authorized by the WHO. The ICD-10 Symptom Rating (ISR) is a brand-new 29-item self-rating questionnaire containing scales for evaluating eating disorders, OCD, depression, and anxiety [16]. The three-page Patient Health Questionnaire (PHQ9) is completely self-administered by the patient. The PHQ evaluates eight diagnoses, broken down into subthreshold disorders (disorders whose criteria encompass fewer symptoms than are required for any specific DSM-IV diagnoses: other depressive disorder, probable alcohol abuse/dependence, somatoform, and binge eating disorder) and threshold disorders (disorders that correspond to specific DSM-IV diagnoses: major depressive disorder, panic disorder, other anxiety disorder, and bulimia nervosa) [17]. Sometimes depressed individuals are not willing to provide reliable information due to the nature of the disorder which can lead to inaccurate diagnosis. So, it is essential to find more effective methods to diagnose depression.

There are also other methods to detect depression effectively. Studies indicate that depressed individuals have abnormal brain activity compared to depression control individuals. Functional magnetic resonance imaging (fMRI) is an imaging technique to investigate brain functionality and structure. Yang et al. has shown results that depression can be identified using fMRI [18]. Electroencephalography (EEG) is a noninvasive technique for evaluating brain function. Using electrodes attached to the scalp, EEG analyzes the electrical activity of sizable groups of synchronously firing neurons in the brain [19]. EEG is used to detect electrical activity of the brain which is being used as a diagnostic biomarker for depression. Depression creates emotional variation and unusual brain activity which can be detected by EEG; thus, EEG can identify depression [20]. MRI can provide more accurate results, but it is not a very practical way of detecting depression. MRI is expensive, can cause claustrophobia, is not portable, and is not easy to use [21]. On the other hand, EEG is portable, noninvasive, easy to use, gives higher temporal resolution, and is less expensive than other brain monitoring methods [22]. With the help of machine learning (ML) and deep learning, the electrical signals recorded from the EEG can be used to classify depressed and depression control individuals. There are existing studies that have shown great promise to diagnose depression using ML. So, ML can diagnose depression by using the EEG signal.

### 1.1. Related Works

In recent years, a lot of research has been conducted to detect depression using EEG signals. Various methods have been proposed for depression detection using EEG signal properties. Various researchers used various screening tools or clinical methods to identify depression among participants.

Mumtaz et al. used DSM IV as the screening tool to identify depression among the participants. They collected EEG data from 64 participants (34 with MDD aged 40.3 ± 12.9 and 30 healthy aged 38.3 ± 15.6) using Brain Master Discovery 24 device. They extracted wavelet, STFT, and EMD features and applied logical regression to classify the data, and acquired 90.5% accuracy with 10-fold CV [23]. The same dataset was used by [24–33]. Here, Mahato and Paul extracted band power, interhemispheric asymmetry, relative wavelet energy, and wavelet entropy as features, multilayered perceptron neural network (MLPNN), and radial basis function network (RBFN) classifier which similarly obtained 93.3% accuracy [24]. Ke et al. used CNN classifier and achieved 98.81% accuracy [25]. Kang et al. removed artifacts using ICA and achieved 98.85% accuracy using CNN classifier at the alpha band [26]. Mahato and Paul extracted band power and theta asymmetry and achieved 88.3% accuracy using SVM [27]. Saeedi et al. extracted band power, WPD, approximate entropy, and sample entropy features and achieved 98.44% accuracy using the Enhanced KNN classifier [28]. Dang et al. used multivariate pseudo-Wigner distribution (MPWD), novel frequency-dependent multilayer brain (FDMB) features, and CNN to achieve 97.27% accuracy [29]. Movahed et al. extracted statistical and spectral wavelet functional connectivity and nonlinear features and acquired 99% accuracy using SVM with RBF kernel [30]. Aydemir et al. extracted 25 features from the discrete wavelet transform coefficients and applied neighborhood component analysis (NCA) for feature selection. They achieved 99.11% accuracy using Weighted KNN and 99.05% accuracy using the Quadratic SVM classifier [31]. Loh et al. applied CNN to achieve 99.25% accuracy [32]. Movahed et al. extracted critical distance, synchronization likelihood features to achieve 99% accuracy using the Label Consistent KSVD algorithm [33]. Li et al. used an EEG dataset consisting of 14 depressed and 14 healthy participants. The DSM-IV was used as the screening tool to identify depressed participants. The EEG data were recorded using HydroCel Geodesic Sensor Net (HCGSN) device. They have extracted power spectral density (PSD), Hjorth activity, and azimuthal equidistant projection (AEP) for image classification. They achieved 89.02% accuracy by extracting PSD features from the full band with an SVM classifier. And they have achieved 84.75% accuracy at the alpha band using CNN classifier [34]. Li et al. in the year 2019 [35] and 2020 [36] used a dataset containing EEG data of 24 depressed and 24 healthy participants. The depression detection was performed using the BID-II screening tool and the EEG data was recorded using HydroCel Geodesic Sensor Net (HCGSN) device. Li et al. in year 2019 achieved 85.62% accuracy by filtering the data from 1 to 40 Hz and using computer-aided detection (CAD) system using a convolutional neural network (ConvNet) with 24-fold CV [35]. Li et al. in the year 2020 used 3 channels of data and filtered the data from 0.5 to 70 Hz. They achieved 80.74% accuracy using CNN classifier [36]. Akbari et al. acquired data from 44 participants (22 depressed and 22 healthy) aged between 23 and 58 years. By extracting the rhythm feature by empirical wavelet transform and centered correntropy features with SVM classifier, 98.33% accuracy was achieved using FP1-T3 channels data and 98.76% accuracy was achieved using FP2-T4 channels data [37]. Then, using reconstructed phase space of the EEG and genetic algorithm (GA) with SVM classifier, 97.74% accuracy was achieved using FP1-T3 channels data and 99.3% accuracy was achieved using FP2-T4 channels data [38]. Then, they collected fluctuation index as features and cascade forward neural network (CFNN) as a classifier which achieved 99.5% accuracry using FP1-T3 channels data and 100% accuracy using FP2-T4 channels data [39]. Cai et al. used a dataset containing EEG data of 92 depressed and 121 healthy participants. PHQ9 was used as a screening tool to identify depression among the participants. They extracted peak, variance, Hjorth parameter, skewness, kurtosis, relative centroid frequency, absolute centroid frequency relative power, absolute power, Kolmogorov Entropy, Shannon Entropy, co-complexity, correlation dimension, and power-spectral entropy as features and achieved 79.27% accuracy using KNN classifier [20]. Cai et al. used a dataset containing EEG data of 152 depressed and 113 healthy participants aged between 18 and 55 years. They extracted features such as variance, peak, kurtosis, inclination, Hjorth parameter, co-complexity, correlation dimension, power spectrum entropy, Kolmogorov entropy, and Shannon entropy. For classification, they used decision tree (DT) and achieved 76.4% accuracy [40]. Cai et al. in the year 2020 used a dataset containing EEG data of 86 depressed and 92 healthy participants aged between 18 and 55 years. Here, PHQ9 was used as the screening tool. They extracted linear features (band powers, center frequency, skewness, kurtosis, and peak of the whole band) and nonlinear features (variance, Hjorth's activity, power spectral entropy, Kolmogorov entropy, Shannon entropy, correlation dimension, and co-complexity). Using the KNN classifier, they achieved 86.98% accuracy [41]. Wu et al. used an EEG dataset consisting of data from 24 depressed (aged 29.7 ± 10.9) and 31 healthy (aged 29.75 ± 9.9) participants. The BDI-II and DSM-IV were used as screening tools for depression detection and HydroCel Geodesic Sensor Net (HCGSN) device was used for EEG recording. They extracted spectral power density (SPD) and the band power (BP) and achieved 83.64% using conformal kernel SVM (CK-SVM) as a classifier [42]. Acharya et al. used a dataset that contains EEG data from 15 depressed and 15 healthy individuals aged between 20 and 50 years. They applied CNN classifier and achieved 93.54% accuracy using FP1-T3 channels (left hemisphere) data and 95.49% accuracy using FP2-T4 channels (right hemisphere) data [43]. Kim et al. used 30 depressed (aged 42.5 ± 16.96) participants' EEG data and 37 healthy (aged 29.75 ± 9.9) participants' EEG data for their research. They extracted four types of electrodermal activity features (dMSCL, dSDSCL, dSKSCL, and dNSSCR). They achieved 74% accuracy by applying the extracted features to a support vector machine recursive feature elimination (SVM-RFE) for feature selection and decision tree (DT) classification [44]. Bachmann et al. used an EEG dataset of 26 participants (13 depressed and 13 healthy) aged between 18 and 66. The ICD-10 screening tool was used to identify depression among participants and Neuroscan Synamps2 was used to record EEG signals. They used alpha band power variability, relative gamma power, spectral asymmetry index, Lempel-Ziv complexity, detrended fluctuation analysis, and Higuchi's fractal dimension as features which they applied to the logistic regression classifier and achieved 92% accuracy [45]. Arora et al. used EEG data from 25 participants aged between 16 and 60 years. The DSM-IV was used as the screening tool. For features, they measured correlation dimension and co-complexity then applied the features to an SVM classifier with RBF kernel and got 91% accuracy [46]. Ay et al. used a dataset containing EEG data from 15 depressed and 15 healthy participants aged between 20 and 50 years. They applied CNN classifier to achieve 97.66% accuracy using FP1-T3 channels (left hemisphere) data and 99.12% accuracy using FP2-T4 channels (right hemisphere) data [47]. Peng et al. worked with a dataset with 27 depressed (aged 31.67 ± 10.94) and 28 healthy (aged 31.82 ± 8.76) participants with EEG data. The PHQ9 was used as a screening tool for depression detection and HydroCel Geodesic Sensor Net (HCGSN) device was used for EEG recording. They extracted phase lag index (PLI) and high discriminative power features. They achieved 92% accuracy by applying SVM with the linear kernel as a classifier [48]. Mohammadi et al. worked with EEG data from 60 participants (aged 32.4 ± 10.5). The participants were evaluated for depression using DSM-IV and BDI-II screening tools. They extracted fuzzy entropy, Katz fractal dimension, and fuzzy fractal dimension features, and then achieved 90% accuracy using fuzzy function based on neural network (FFNN) [49]. Wan et al. worked with 2 datasets. The first dataset contains EEG data from 35 participants aged between 20 and 56 years and the second dataset contains EEG data from 30 participants aged between 24 and 55 years. The DSM-IV and HAM-D were used as screening tools to evaluate depression among participants. For the features, they extracted wavelet features, power spectral entropy, co-complexity, approximate entropy, and wavelet entropy. Using the KNN classifier with a genetic algorithm for feature selection, they achieved 94.29% accuracy for the first dataset and by using the regression trees classifier with a genetic algorithm, they achieved 86.67% accuracy for the second dataset [50]. Zhu et al. used a dataset with EEG signals from 19 depressed (aged 21.1 ± 1.95) and 20 healthy (aged 20.11 ± 2.07) participants. The BDI-II was used as the screening tool and the data was recorded using HydroCel Geodesic Sensor Net (HCGSN) device. They extracted variance, maximum power, sumpower, approximate entropy, Kolmogorov entropy, permutation entropy, Lempel–Ziv complexity, correlation dimension, Lyapunov exponent, singular-value deposition entropy, min-entropy, Shannon entropy, spectral entropy, Hartley entropy, co-complexity as features with BestFirst algorithm for feature selection. They achieved 83.42% accuracy by using an SVM classifier with a linear kernel [51]. Thoduparambil et al. worked with a database taken from the Public Domain Dedication and License (PDDL) v1.0; the data was recorded using a Neuroscan Synamps2 system. They applied CNN classifier and achieved 98.84% accuracy from the channels located at the left part of the brain and 99.07% accuracy from the channels located at the right part of the brain [52]. Čukić et al. used EEG data collected from 21 depressed and 20 healthy participants aged from 24 to 68 years. The depression was identified by ICD-10 screening tools and EEG was recorded using NicoletOne Digital EEG Amplifier. They extracted Higuchi's Fractal Dimension (HFD) and Sample entropy. They achieved 97.57% accuracy using multilayer perceptron, logistic regression, decision tree, and Naïve Bayes classifier [53]. Mahato et al. worked with EEG data collected from 24 depressed (aged 35 ± 5.9) and 20 healthy (aged 36 ± 4.2) participants. The DSM-V and HAM-D were the screening tools used to identify depression. They extracted band power, interhemispheric asymmetry, paired asymmetry, sample entropy, and detrended fluctuation analysis as features and achieved 96.02% accuracy with SVM as a classifier [54]. Liu et al. worked with a dataset containing EEG data from 20 depressed and 19 Healthy participants aged between 23 and 65 years. For screening depression among participants, HAM-D was used, and EEG data were recorded using a Neuroscan Quik-cap device. They measured quantified influence, phase synchronization, and functional integration, including degree, functional segregation, clustering coefficient, and characteristic path length, and did statistical analysis from the data and used PCA for feature selection. They achieved 89.7% accuracy for the beta band using the SVM classifier [55]. Bai et al. used a dataset containing EEG data from 142 depressed and 71 healthy participants. They extracted absolute centroid, variance, relative power, absolute power, power spectral density, activity, skewness, kurtosis, spectral entropy, Higuchi's fractal dimension, Hjorth parameters, and detrended fluctuation analysis as features and achieved 81.16% accuracy using tree-based feature selection and random forest classifier [56]. Uyulan et al. used EEG data collected from 46 depressed and 46 healthy participants aged between 20 and 51 years. The HAM-D was used as the screening tool to identify depression among participants and Neuroscan/Scan LT was used to record EEG data. Using CNN with MobileNet architecture, they achieved 89.33% from the left hemisphere and 92.66% from the right hemisphere. They have also achieved 90.22% accuracy in the delta band using CNN with ResNet-50 architecture [57]. Avots et al. used EEG data collected from 20 participants (aged between 24 and 60 years) using the Cadwell Easy II EEG device. The HAM-D was used for screening purposes. They extracted alpha power variability, relative band power, spectral asymmetry index, Lempel–Ziv complexity, Higuchi fractal dimension, and detrended fluctuation analysis as features. Using ReliefF for feature selection, they achieved 95% accuracy with both KNN and decision tree classifier [58]. Lei et al. worked with EEG data collected from 101 depressed participants, 82 participants with bipolar disorder, and 81 healthy participants using the Brain Products GmbH device. Using the CNN classifier, they achieved 96.88% accuracy with depressed vs. healthy and 97.3% accuracy with bipolar vs. healthy [59]. Zhao et al. used EEG data collected from 40 depressed participants and 38 healthy participants (aged 18.72 ± 0.36) using a device from Neuroscan. The BDI-II was used for screening depression. Microstate and Omega complexity features were extracted and using SVM and they achieved 76% accuracy [60]. Liu et al. worked with 2 datasets. The first dataset contains EEG data collected from 24 depressed and 29 healthy participants using HydroCel Geodesic Sensor Net (HCGSN) and the second dataset contains data collected from 16 depressed and 16 healthy participants. For the first dataset, PHQ9 was used for screening, and for the second dataset, BDI was used for screening. They achieved 89.63% accuracy from the first dataset and 88.56% accuracy from the second dataset using CNN classifier with gated recurrent unit (GRU) [61]. Nassibi et al. worked with EEG data collected from 42 depressed (aged 18.64 ± 1.12) and 42 healthy (aged 19.04 ± 1.16) participants using Neuroscan Synamps2. The screening was performed using BDI-II. They extracted band power, relative band power, maximum Power spectral density, power spectral density, median frequency, relative median, mean frequency, Shannon entropy, Hjorth parameters, root-mean-square, kurtosis, skewness, variance, and singular value as features and neighborhood component analysis (NCA) for feature selection. Using the Naïve Bayes classifier, they achieved 91.8% accuracy [62]. Seal et al. used EEG data collected from 46 depressed and 46 healthy participants (aged between 20 and 51 years) using EEG Traveler Braintech 32+ CMEEG-01. The PHQ9 was used for screening. They extracted band power, mean, median, mode, mean cube, standard deviation, first difference, normalized first difference, second difference, normalized second difference, mobility, Pearson's coefficient of skewness, Shannon entropy, Alpha asymmetry 1, and Alpha asymmetry 2 as features and ANOVA test and correlation analysis for feature selection. They achieved 87% accuracy using the XGBoost classifier [63].

### 1.2. Contribution and Objectives

We can observe that previously multiple research work has been performed using both machine learning and deep learning algorithms. We have noticed that over the years most of the datasets that have been created had participants of all ages. No dataset was created using young adults (aged 18–25 years). And if we investigate the depression screening tools, we can see that there is a hand full of work which has been performed with PHQ9 which is a very good self-administrable screening method for depression. In previous works, the EEG recording devices that have been used were bulky, wired, and not easy to use, which may not serve as an ideal alternative for depression diagnosis from traditional methods. EEG-based depression detection should provide a better and more accurate diagnosis and also should be easy to use, so that the patients who are not willing to face questioner/interview base diagnosis can have a better, more reliable, and easy solution.

We want to create an EEG-based depression detection technique that will be reliable and easy to use. For this, we want to target young adults aged between 18 and 25 years (University students). Use the PHQ9 screening tool for a depression diagnosis. Use the Emotiv EPOC+ EEG headset which is wireless and easy to use for data recording. Create a machine learning model which will be reliable and will give the best results.

## 2. Materials and Method

In this section, we will discuss the proposed method. The proposed method can be divided into a few parts. The flowcharts of the proposed method are shown in the following figures from Figures 1–9.

### 2.1. Dataset Acquisition

**Figure 1:**
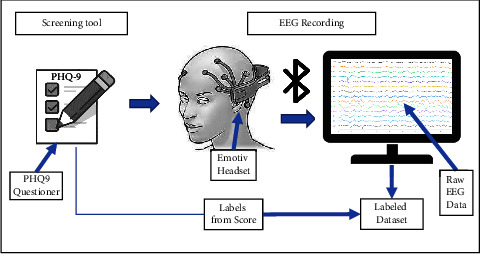
Data acquisition using Emotiv Epoc+ and PHQ9 screening tool.

**Figure 2:**
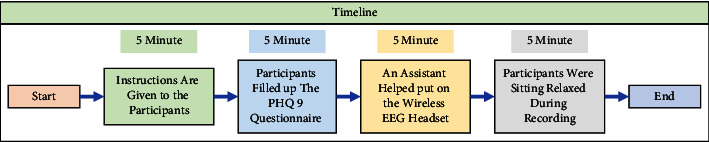
Timeline for each recording session.

The target of this work is to identify depression among young adults. Initially, there was a survey to identify depressed and depression control participants aged between 18 and 25 years. Over 500 students participated in the initial survey. All the participants were university students from Independent University, Bangladesh (IUB). The Patient Health Questionnaire (PHQ9) was used in the initial survey to select depressed and depression control individuals. After the survey, 82 participants were selected to record EEG data. But only 64 participants were willing to give consent for EEG recording. The recording was performed a few weeks after the survey. All participants signed consent forms for the recording. For labeling purposes, the participants were asked to fill up the PHQ9 questionnaires before the EEG recording. During the recording, participants were sitting in a comfortable chair and were asked to sit still and to keep their eyes closed during the EEG recording.  Figure 1 shows the flow chart for data acquisition. The recordings were 5 min long for each participant.  Figure 2 shows the timeline of each recording session. This shows that a total of 20 min (Approx.) was required for every session for each participant. Depending on the PHQ9 score 32 participants (16 males and 16 females, aged between 18 and 25 years) recordings were chosen for this work. Among the 32 participants, 19 were identified as depressed (age 21.6 ± 1.98) and 13 were identified as depression control participants (age 21.3 ± 2.06). Here, we have observed that among the depressed group, 74% were female participants. This also supports the WHO report that depression is more common among females than males [1].

Psychiatrists or mental health care centers typically favor the PHQ9-based depression screening approach. The entire exercise takes between two and five minutes to complete.  Table 1 displays the severity measuring score and its accompanying labels.

For this study, we have only considered the participants who scored between 20 and 27 were selected as the depressed group, and the participants who scored between 0 and 4 were selected as the control group. The information on the participants is given in Table 2.

The dataset was recorded using Emotiv EPOC+ 14-channel EEG Device and the sampling frequency was 128 Hz. Emotiv is a low-cost, wireless, and portable device. The channels are placed according to the 10–20 system. There are eight frontal electrodes (AF3, F3, F7, FC5, AF4, F4, F8, and FC6), two temporal electrodes (T7 and T8), two parietal electrodes (P7 and P8), two occipital electrodes (O1 and O2), and two reference channels (P3 and P4).  Figure 6 shows the channel locations, Emotiv Eopc+ headset, and the list of channels located at different brain regions.

**Figure 4:**
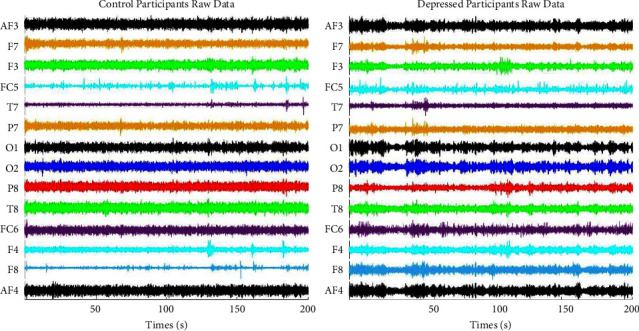
Raw data from control and depressed group.

**Figure 5:**
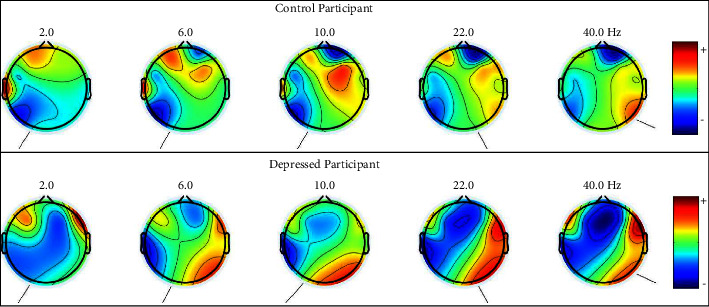
Brain maps of control and depressed participants at different frequency points.

There are 14 EEG signals (From each channel) collected from each participant. 266 EEG signals from depressed participants (19 Participants × 14 Channels) and 182 EEG signals from depression control participants (13 Participants × 14 Channels). Here, a total of 448 EEG signals have been recorded for this work.  Figure 4 shows EEG data collected from depression control and depressed participants.  Figure 5 shows the brain maps of the depression control and depressed participants at 2 Hz, 6 Hz, 10 Hz, 22 Hz, and 40 Hz. Each of the frequency points represents a single point of different sub-bands (Delta, Theta, Alpha, Beta, and Gamma). And a significant difference can be observed from the Brain Maps.

### 2.2. Segmentation

From each subject, 5 min of data was recorded. So, we collected 32 samples which were not enough for machine learning. Therefore, segmentation was required. Segmentation helps create smaller samples from a big sample. We carefully divided the raw data of each participant into multiple nonoverlapping data with their corresponding channels. Each 5 min data was segmented into 1, 2, 3, 4, and 5 seconds creating 5 datasets with different sample lengths. Each segment contains an EEG signal from 14 channels.  Table 3 contains the total number of samples and the group-wise number of the sample after dividing the raw data into different segments.

If we consider *P* equal to the total number of samples collected from participants, *i* represents the number of participants, *T* is the total number of samples in each recording, *S* is the segment length, *C* is the number of channels, and *N* is the number of samples after segmentation, then

(1) $$\begin{array}{c} N=\frac{T}{S}\text{,} \end{array}$$

(2) $$\begin{array}{c} P\left(i\right)=T\times C=N\times S\times C\text{,}\\ i=1,2,\ldots 32\text{.} \end{array}$$

We have a total of 5 min data equivalent to 38400 samples (sampling 128 Hz); now if we follow equation (2), we can calculate the total number of samples we can get from each recording for desired segment length. Now if we chose 5 sec (640 samples) as segment length, then we will get 60 samples after segmentation. So, from each participant's 5 mins data of 38400 × 14 samples, we will be equal to 60 × 640 × 14 samples for choosing a 5-sec segment length by following equations (1) and (2).

### 2.3. Preprocessing

**Figure 6:**
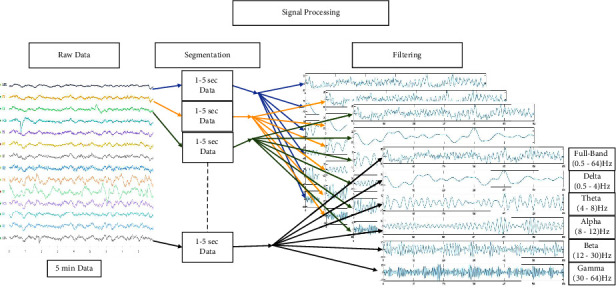
Segmentation and signal processing.

**Figure 7:**
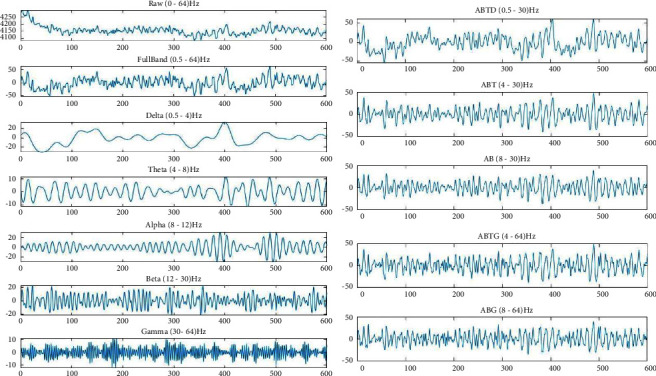
Filtered data at different frequencies.

We applied IIR Butterworth filters to all the channels for preprocessing the data. To find the optimal frequency band for the best result, we have filtered the raw data into multiple sub-bands. The signal-processing steps are shown in Figure 6. We have filtered the raw data to acquire 0.5–64.0 Hz for the full band, 0.5–4.0 Hz for the Delta band, 4.0–8.0 Hz for the Theta band, 8.0–12.0 Hz for the Alpha band, 12.0–30.0 Hz for the Beta band, 30.0–64.0 Hz for Gamma band, and some additional filtering by excluding one or few frequency bands. We have finally acquired 0.5–30.0 Hz (ABDT) excluding Gamma, 4.0–64.0 Hz (ABTG) excluding Delta, 4–30.0 Hz (ABT) excluding Delta, and Gamma, 8.0–30.0 Hz (AB) excluding Delta, Theta, and Gamma, 8.0–64.0 Hz (ABG) excluding Delta and Theta as different frequency bands for further analysis.  Figure 7 shows the filtered data.

### 2.4. Feature Extraction

**Figure 8:**
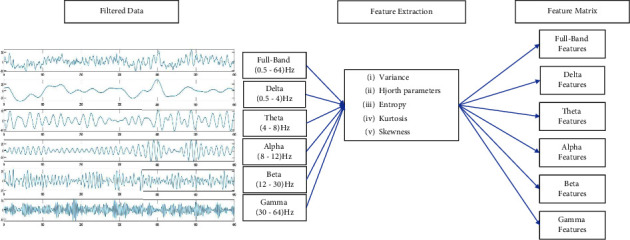
Creating feature matrix by extracting features from filtered data.

Depending on the characteristics of EEG signals, linear or nonlinear features can be extracted. For this study, we have extracted features such as variance, Hjorth activity, Hjorth mobility, Hjorth complexity, kurtosis, skewness, Shannon entropy, and Log energy entropy to create feature matrixes (shown in Figure 8). The features were applied to the classifier individually and combined to get the best possible features.

#### 2.4.1. Variance

Basically, variance is a statistical term that describes how data are distributed relative to their mean or expected value. It is the only kind of probability distribution that accounts for the degree of dispersion of a set of data.

#### 2.4.2. Hjorth Parameters

Hjorth parameters consist of activity, mobility, and complexity. Hjorth parameters are linear features that are obtained in the time domain by applying various signal-processing methods.

(i) *Hjorth activity*: This represents the variance of a time function as well as the signal power. The activity provides a measurement of the squared standard deviation of the time domain signal's amplitude. This may represent the frequency domain power spectrum's surface. The activity will be depicted by the following equation, where *x* is a signal and *t* is the time:
  (3) $$\begin{array}{c} Hjorth\;Activity=Var\left(x\left(t\right)\right)\text{.} \end{array}$$
(ii) *Hjorth mobility*: The mobility parameter represents the power spectrum's mean frequency or standard deviation as a percentage. Equation (4) represents Hjorth mobility, where it is determined by dividing the square root of the variance of the signal's *x* (*t*) first derivative by the variance of the signal *x* (*t*).
  (4) $$\begin{array}{c} Hjorth\;Mobility=\sqrt{\frac{Var\left(dx\left(t\right)/dt\right)}{Var\left(x\left(t\right)\right)}}\text{.} \end{array}$$
(iii) *Hjorth complexity*: Complexity provides an estimate of the signal's bandwidth and shows how a signal resembles a pure sine wave in terms of shape. Equation (5) represents Hjorth mobility. It is described as the proportion of the time derivatives of the mobility of the signal *x* to the mobility signal *x* at time *t*.
  (5) $$\begin{array}{c} Hjorth\;Complexity=\frac{Mobility\left(x\left(t\right)/dt\right)}{Mobility\left(x\left(t\right)\right)}\text{.} \end{array}$$

#### 2.4.3. Entropy

A random process's degree of uncertainty can be gauged using entropy. It represents the signal's unpredictability. Without failure, rolling element equipment often produces a more random signal, but machines with failure typically produce a more predictable signal. Entropy is thought to be a powerful characteristic for identifying emotions in EEG signals. In this study, Shannon entropy and Log energy entropy were used.

(i) *Shannon entropy*: Shannon Entropy is a metric for the unpredictability of a random variable and a random signal. The uncertainty and randomness increase with increasing entropy. It can be represented by the following equation:
  (6) $$\begin{array}{c} ShnEn=-\overset{N}{\underset{i=1}{\sum }}p\left(x_{i}\right)\log\left(p\left(x_{i}\right)\right)\text{,} \end{array}$$
where *p*(*x*_*i*_) is the probability of *i* number sample of the signal *x* and *N* denotes the length of the signal.
(ii) *Log energy entropy*: Log energy (LogEn) entropy is related to the energy of the signal. It is similar to wavelet entropy, but only uses the summation of logarithms of the probabilities. It is used to analyze the EEG signal's complexity. It can be defined by the following equation.
  (7) $$\begin{array}{c} LogEn=\overset{N}{\underset{i=1}{\sum }}\log\;\left(x_{i}^{2}\right)\text{.} \end{array}$$
Here, *x*_*i*_ is the *i* number sample of the signal and *N* denotes the length of the signal.

#### 2.4.4. Kurtosis

Kurtosis can measure the peakedness of an EEG signal. When the signal has a normal distribution, the kurtosis will be three and when the signal will not have normal distribution the kurtosis will be higher than three (for heavier peak) or less than three (for lighter peak). If the signal is *x*, the mean of the signal is $\bar{x}$, and length is *N*, then kurtosis can be defined by the following equation:

(8) $$\begin{array}{c} Kurt=\frac{\left(1/N\right)\overset{N}{\underset{i=1}{\sum }}{\left(x_{i}-\bar{x}\right)}^{4}}{{\left(\left(1/N\right)\sum_{i=1}^{N}{\left(x_{i}-\bar{x}\right)}^{2}\right)}^{2}}-3\text{.} \end{array}$$

#### 2.4.5. Skewness

Skewness is a metric for a distribution's asymmetry. When the left and right sides of a distribution are not mirrored, the distribution is said to be asymmetrical.

(9) $$\begin{array}{c} Skew=\frac{\left(1/N\right)\overset{N}{\underset{i=1}{\sum }}{\left(x_{i}-\bar{x}\right)}^{3}}{{\left(\left(1/N\right)\sum_{i=1}^{N}{\left(x_{i}-\bar{x}\right)}^{2}\right)}^{\left(3/2\right)}}\text{.} \end{array}$$

Here, *x*_*i*_ is the *i* th number sample of the signal, $\bar{x}$ is the mean of the signal, and *N* denotes the length of the signal.

### 2.5. Classification

**Figure 9:**
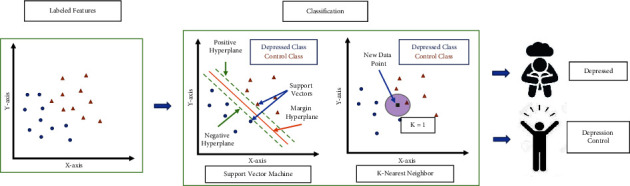
Classifying depressed and depression control participants using SVM and KNN.

For classification, we used support vector machine (SVM) algorithms and K-nearest neighbor (KNN) algorithms (shown in Figure 9). Linear, quadratic, cubic, and Gaussian radial basis are the kernels we used for SVM, and fine KNN, medium KNN, coarse KNN, cosine KNN, cubic KNN, and weighted KNN are the different types of KNN classifiers we used to identify the best option for the project. The description of the classifiers is provided in Table 4. It is difficult to know which classifier will give the best outcome as not all data are the same. Our dataset is new and recorded using an Emotiv EPOC+ headset, unlike other depression-related datasets. So, we decided to apply multiple classifiers to find out which classifier will be the best for our dataset.

### 2.6. Experiments

We conducted several experiments for this research work. First, we extracted all the features from the full band (0.5–64 Hz) of different sample lengths and fetched the features to the classifiers separately and combined them to find the best features and sample length. After selecting the sample length and the features, we extracted those features from different frequency bands and fetched them to the classifiers with 25 iterations to select the frequency range and the best classifier for this work. We used five-fold cross-validation for the validation. Cross-validation is very important as it tests the performance of a machine learning algorithm to classify new data and prevents problems such as overfitting.

**Figure 10:**
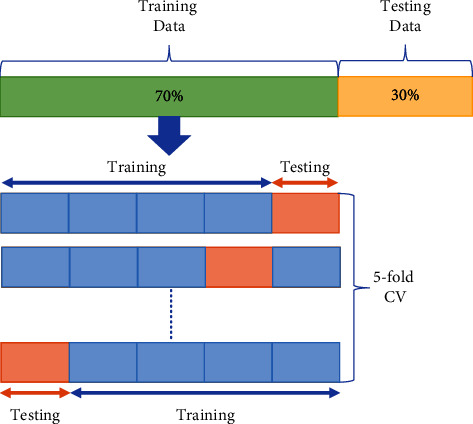
Data splitting and cross-validation.

After that, we extracted features from different channels located at the different regions of the brain (left hemisphere, right hemisphere, frontal lobe, parietal lobe, temporal lobe, and occipital lobe) from the selected frequency range and classified the data using the selected classifier to determine which channels give the best outcome. For this part, we used 70 percent of the data for training, for validation we used five-fold cross-validation on 70 percent of the data, and the remaining 30 percent of the data for classification (shown in Figure 10). We used 10 iterations for all classifications and each time the training testing data were selected randomly.

### 2.7. Performance Evaluation

To evaluate the performance of the proposed experiments we considered several performance parameters. We have considered accuracy, precision, negative predictive value (NPV), sensitivity, specificity, and F1 score.

#### 2.7.1. Confusion Matrix

**Figure 11:**
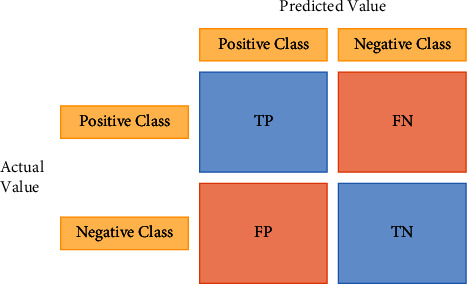
Confusion matrix.

In the field of machine learning algorithms, a confusion matrix is a matrix or table that helps summarize and visualize the performance of a classification algorithm. It is an *n*-by-*n* matrix where we can see the true and false predictions of a classification algorithm. We can get the number of true positives (TP), true negatives (TN), false positives (FP), and false negatives (FN) from the confusion matrix (FN). Here, TP stands for the correct forecasts of the positive class, TN for the accurate predictions of the negative classes, FP for the positive class's incorrect predictions, and FN for the wrong predictions of the negative classes (shown in Figure 11).

#### 2.7.2. Accuracy

It gives us an idea that how many times the classification algorithm was able to predict correctly. It can be calculated using the following equation:

(10) $$\begin{array}{c} Accuracy=\frac{TP+TN}{TP+TN+FP+FN}\text{.} \end{array}$$

#### 2.7.3. Precision

Precision is also known as the positive predictive value (PPV). It is a performance parameter of an ML algorithm that tells us the performance of a positive prediction made by the algorithm. The precision is calculated by equation (11). Here, the total number of true positives is divided by the sum of true positives and false positives.

(11) $$\begin{array}{c} Precision=\frac{TP}{TP+FP}\text{.} \end{array}$$

#### 2.7.4. Negative Predictive Value

Negative Predictive Value (NPV) represents the possibility of negative predicting being negative. The NPV is calculated by equation (12). Here, the total number of true negatives is divided by the total number of negative predictions.

(12) $$\begin{array}{c} NPV=\frac{TN}{TN+FN}\text{.} \end{array}$$

#### 2.7.5. Sensitivity

It is also known as recall or true positive rate (TPR). When the classification is binary, the sensitivity is known as recall. It represents how well a classification algorithm can predict truly positive cases. It is calculated by dividing the total number of true positives by the sum of all true positives and false negatives, as follows:

(13) $$\begin{array}{c} Sensitivity=\frac{TP}{TP+FN}\text{.} \end{array}$$

#### 2.7.6. Specificity

It is also known as selectivity or true negative rate (TNR). It represents how well a classification algorithm can predict truly negative cases. It is calculated as the total number of true positives divided by the total number of true positives and false negatives (shown in equation (14)).

(14) $$\begin{array}{c} Specificity=\frac{TN}{TN+FP}\text{.} \end{array}$$

#### 2.7.7. F1 Score

It is a measure of the classification accuracy of a binary classifier. By utilizing equation (15), F1 score is calculated. The harmonic mean of sensitivity and specificity is the F1 score.

(15) $$\begin{array}{c} F1=\frac{2TP}{2TP+FP+FN}\text{.} \end{array}$$

#### 2.7.8. Implementation

The experiments were conducted using MATLAB (2020A). MATLAB was installed on a laptop with Intel (R) Core (TM) i7 CPU with 16 GB RAM and NVIDIA GeForce GTX 1650 GPU.

## 3. Results and Discussion

**Figure 12:**
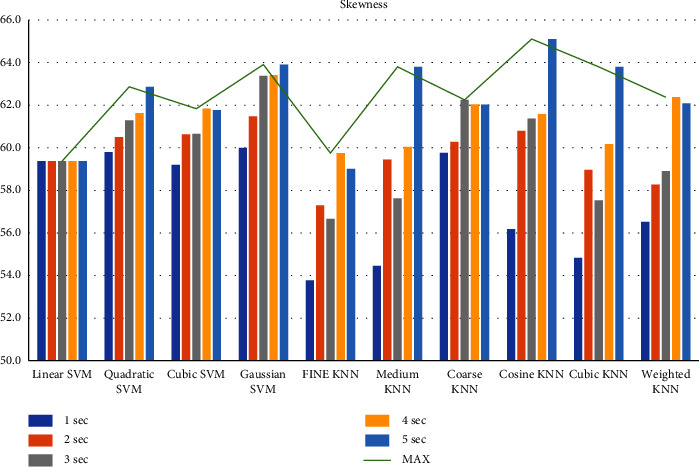
Classification accuracies at different sample lengths using skewness feature.

**Figure 13:**
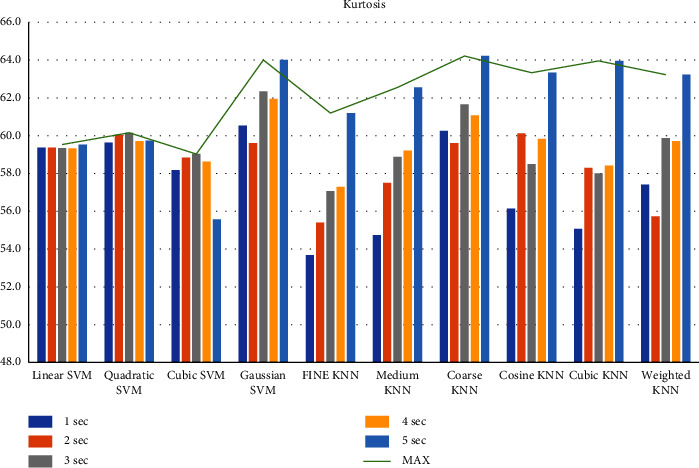
Classification accuracies at different sample lengths using kurtosis feature.

**Figure 14:**
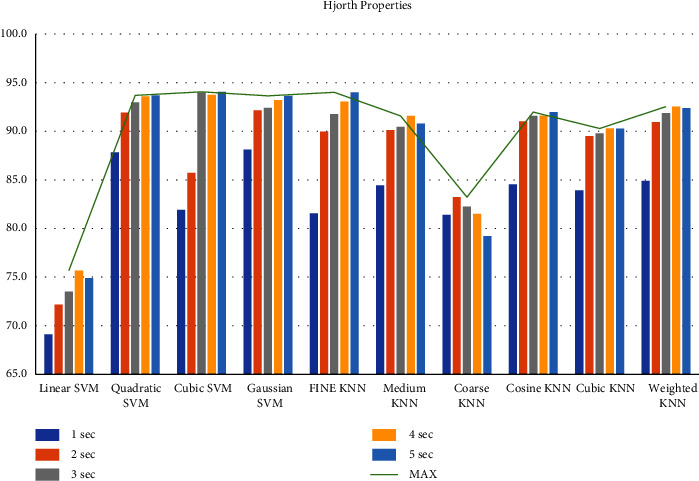
Classification accuracies at different sample lengths using Hjorth parameters.

**Figure 15:**
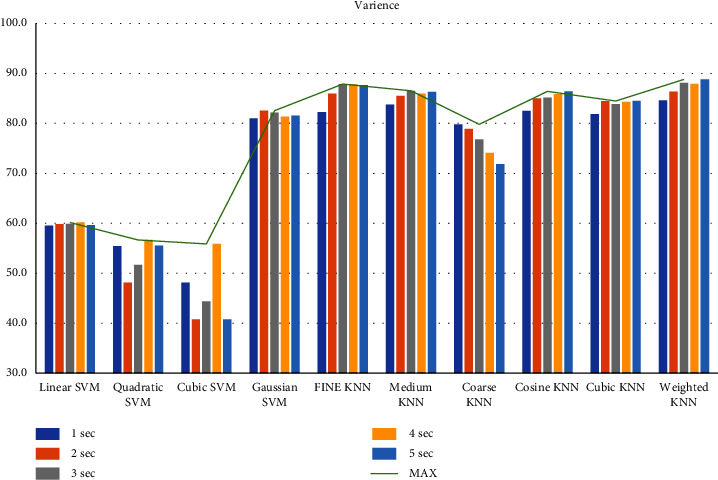
Classification accuracies at different sample lengths using variance feature.

**Figure 16:**
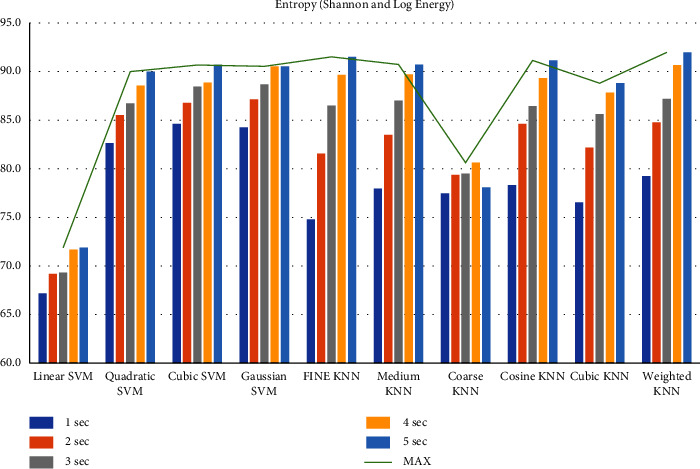
Classification accuracies at different sample lengths using Shannon entropy and log energy entropy features.

**Figure 17:**
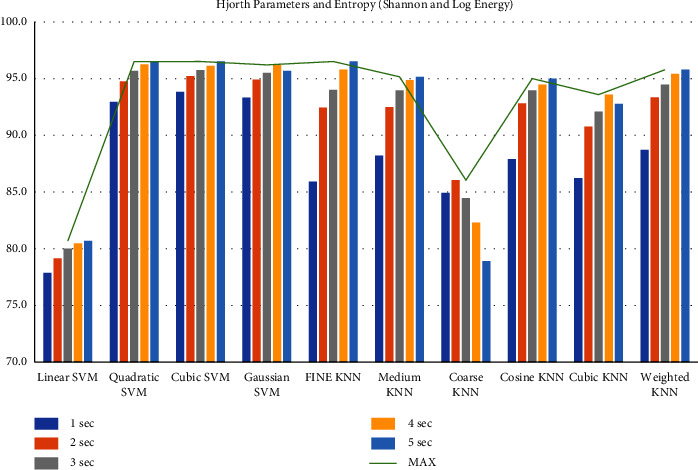
Classification accuracies at different sample lengths using Hjorth parameters, Shannon entropy, and Log energy entropy features.

**Figure 18:**
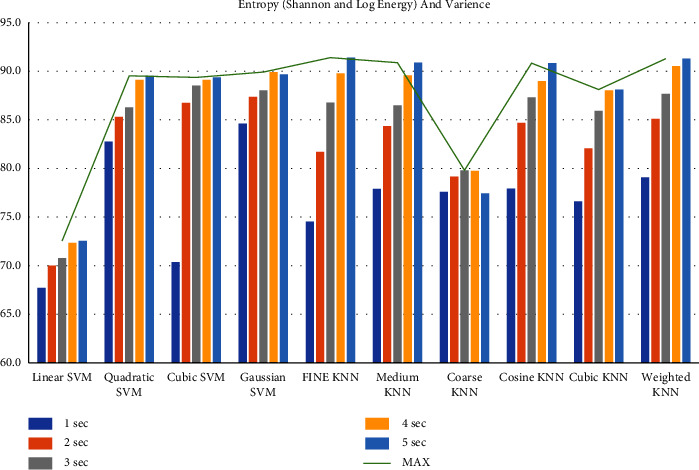
Classification accuracies at different sample lengths using Shannon entropy, Log energy entropy, and variance features.

**Figure 19:**
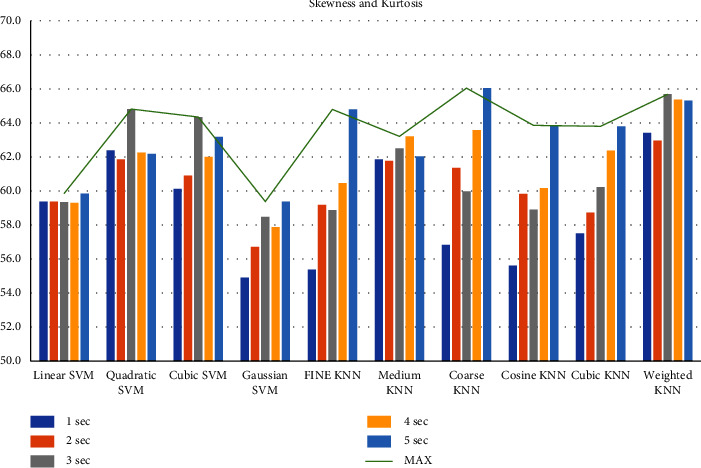
Classification accuracies at different sample lengths using skewness and kurtosis features.

**Figure 20:**
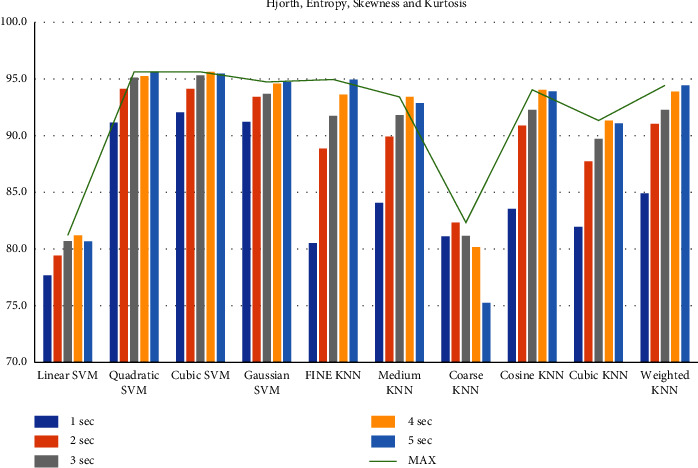
Classification accuracies at different sample lengths using all features.

First, we filtered the raw data at 0.5–64 Hz (full-band) from the different segments (1–5 sec data). Then, we extracted the features to create the feature matrix. The feature matrix then was fetched to the classifiers for classification. In this part, we used a 5-fold CV. The findings from different feature matrices can be seen in “Figures 12–20.” Here we can observe that the individual figures represent the accuracy level of different feature combinations from different classifiers at different sample lengths. Figures 12 and 13 are showing results for skewness and kurtosis features. Both features have poor results at different sample lengths and with all the classifiers. But Hjorth parameters (Figure 14), variance (Figure 15), and entropy (Figure 16) show better results. And Hjorth parameters gave the best accuracy of 94% with Fine KNN classifier and 5-sec sample length.

Now, we combined the Hjorth parameters and entropy (Figure 17), variance and entropy (Figure 18), skewness and kurtosis (Figure 19), and all the features except variance (Figure 20) and observe the accuracy at different sample lengths using the classifiers. We did not include the variance when we combined all the features (Figure 20) because the variance is the same as the first Hjorth parameter (Hjorth activity). After investigating all the results, we can see in Figure 17 that the Hjorth parameters and entropy gives the best results among all other combination of features. Here, the highest accuracy is 96.5% using the 5-sec sample length and with quadratic SVM, cubic SVM, and fine KNN classifiers. From all these findings, we can decide that by extracting the Hjorth parameters (activity, mobility, and complexity) and the entropies (Shannon and Log energy) from 5-sec segments, we can achieve the highest accuracy

**Figure 21:**
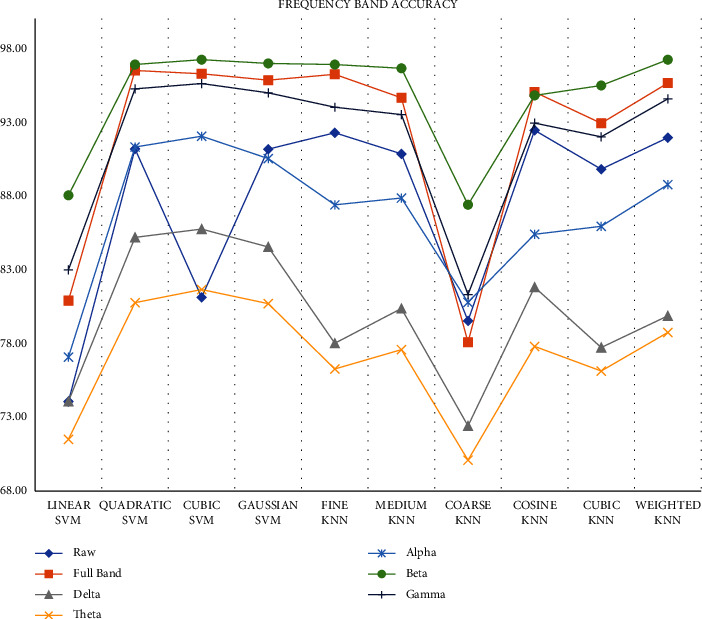
Classification accuracy using different sub-bands.

To further analyze, we extracted the Hjorth parameters and the entropy features from different frequency sub-bands (delta, theta, alpha, beta, and gamma) with 5-sec sample lengths from all channels. Then, we classified the features using all the classifiers with a 5-fold CV and 25 iterations. We calculated the average accuracy and the standard deviation to identify the best classifier and frequency band. From Figure 21, we can observe that the Beta band gives better classification accuracy than the other frequency bands.

Table 5 shows the average accuracy and the standard deviation results. From there we can observe that the beta band with Cubic SVM 97.22 ± 0.21 accuracy and with weighted KNN gives **97.213** **±** **0.18** accuracy. Here, weighted KNN is best as it has a lower standard deviation than Cubic SVM although cubic gives 0.01% higher accuracy. The features of the Beta band give the best accuracy because this band indicates logical thinking and thoughts, and it allows us to focus. A depressed and depression control person will have different thoughts and will have different levels of focus. And therefore, features from the Beta band perform better for depression classification.

**Figure 22:**
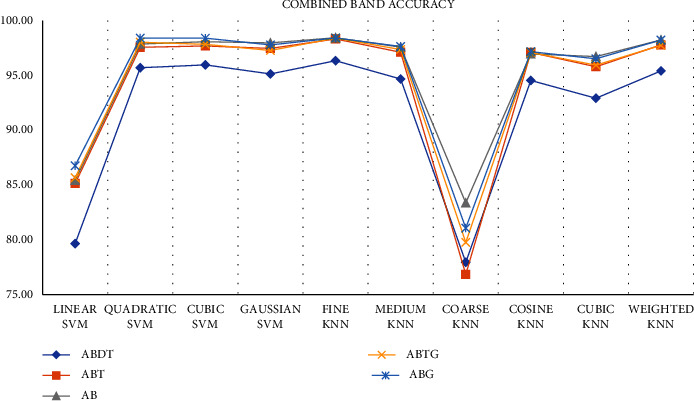
Classification accuracy using different combinations of sub-bands.

After this, we wanted to see if filtering the signal at any other frequency range will improve classification performance or not. So, we filtered the data at different frequencies in a way that the frequency range includes multiple bands. After that, we extracted the features (Hjorth and entropy) from all the filtered data and classified them using all the classifiers. From Figure 22, we can observe the accuracy of the classifiers. Here, we can see that ABDT gives lower accuracy than the others. So, we can exclude that frequency range. But this figure cannot give us a clear picture to choose the best classifier and frequency band.

If we observe the average accuracy and the standard deviation from Table 6, we can see all the bands give better results with fine KNN. Here, ABT, AB, ABTG, and ABG gave accuracy higher than 98% with lower standard deviation. From this experiment, we can decide that fine KNN will perform better with ABT, AB, ABTG, and ABG.

**Figure 23:**
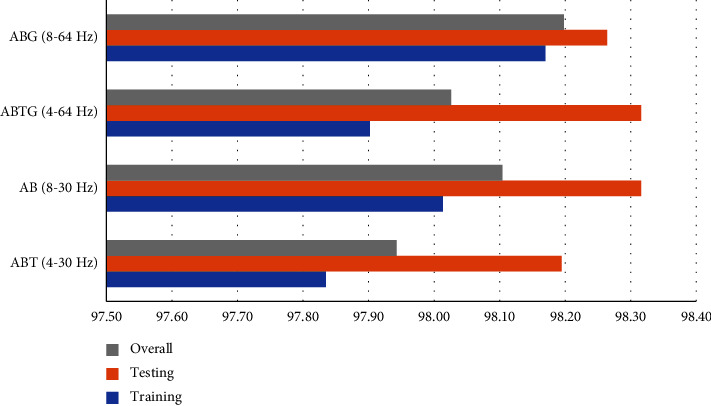
Training, testing, and overall accuracy of different combination of bands using fine KNN classifier.

To check the performance and reliability of ABT, AB, ABTG, and ABG bands with the fine KNN classifier, we measured accuracy, precision, NPV, sensitivity, specificity, and F1 score. For this part, we divided the dataset into a 70/30 ratio for training and testing and we applied a 5-fold CV for training with 10 iterations.  Figure 23 shows the training, testing, and overall average accuracy of the 4 bands. Here, we can observe that ABG gives better training accuracy and AB gives better testing accuracy. And overall ABG accuracy is the highest.

If we observe Table 7, we can see that in terms of overall accuracy, AB has a lower standard deviation (98.10 ± 0.11) and ABG has better accuracy but a high standard deviation (98.20 ± 0.30). Plus, the AB band with fine KNN gives better NPV (0.977 ± 0.002), sensitivity (0.984 ± 0.002), and F1 score (0.984 ± 0.001). And other parameters such as precision (0.984 ± 0.003) and specificity (0.976 ± 0.005) are also satisfactory.

Finally, we can decide that by segmenting the dataset into 5-sec epochs, then filtering the data from 8 to 30 Hz (AB) frequency, extracting the Hjorth parameters (activity, mobility, and complexity) and entropy (Shannon entropy and Log energy entropy) and using fine KNN algorithm, we can create a classifier model that will give the highest accuracy for the dataset we created.

**Figure 24:**
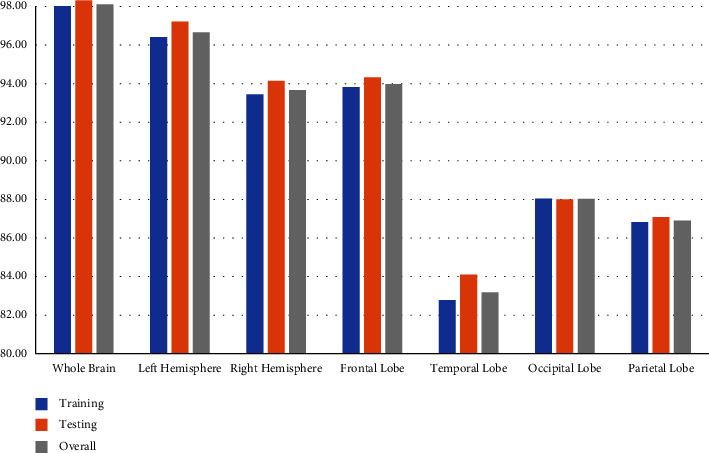
Training, testing, and overall accuracy of different brain regions using fine KNN classifier.

So far, we have used features extracted from all channels (whole brain) to train machine-learning models. Furthermore, we explored different regions of the brain. For this, we created feature matrixes by extracting features from the channels located on the left side of the brain (left hemispheric data), from the channels located on the right side of the brain (left hemispheric data), from the channels located on the frontal lobe of the brain (frontal lobe data), from the channels located on the temporal lobe of the brain (temporal lobe data), from the channels located on the parietal lobe of the brain (parietal lobe data), and from the channels located on the occipital lobe of the brain (occipital lobe data). Then, we compared the classifier performance using those data with data from the whole brain (all channels).  Figure 24 shows the training testing and overall accuracies of the brain regions. And it is clear from the bar chart that the whole brain gives better accuracy compared to other regions of the brain. From the chart, we can also observe that the left hemisphere (96.66 ± 0.45) provides higher accuracy compared to the right hemisphere (93.66 ± 0.35). And frontal lobe (93.97 ± 0.40) gives better accuracy compared to the temporal lobe (83.18 ± 0.53), occipital lobe (88.03 ± 0.35), and parietal lobe (86.90 ± 0.51).

In Table 8, we can also observe that the whole brain gives better results in terms of accuracy, precision, NPV, sensitivity, specificity, and F1 score. So, using whole brain region (all channels data) for depression detection is the best approach for our work.

We can compare our work with the existing work and see the significance of our research.  Table 9 shows the existing research that has been conducted with state-of-the-art methods in recent years with our work.

## 4. Conclusion

In this work, we have recorded EEG data of young adults (19 depressed and 13 Control) evaluated by the PHQ9 screening tool and proposed a machine learning approach to learn about the EEG properties for depression detection.

We conducted multiple experiments with the reported machine learning (SVM and KNN) classifiers with our recorded data. The first experiment we conducted was on segmentation to find the better sample length suitable for ML. From our experiments, we have identified that 5-second segments are suitable for our work. Then, we have identified a suitable frequency range from various experiments that improve performance using features that are related to depression detection. We have found out that Hjorth parameters along with Shannon entropy and long energy entropy provide better results among other reported features and the beta band (12–30 Hz) gives the highest accuracy of 97.21 ± 0.21% with 25 iterations and 5-fold CV using weighted KNN compared to the other sub-bands. By combining the sub-bands, we have also investigated some other frequency ranges. We have found out that by taking the range from alpha to beta 8–30 Hz (AB), we can improve ML performance and achieve 98.43 ± 0.15% accuracy with 25 iterations and a 5-fold CV with fine KNN classifier. Using AB (8–30 Hz), we can see a significant improvement of 1.22% accuracy and slandered deviation. To further investigate the reliability, we divided the dataset 70/30 for training and testing with 5-fold CV and 10 iterations. In this experiment, we have found out that the ML performance is better by choosing the AB (8–30 Hz) band with fine KNN classifier with an accuracy of 98.10 ± 0.11%, precision of 0.984 ± 0.003, NPV of 0.977 ± 0.002, sensitivity of 0.984 ± 0.002, specificity of 0.976 ± 0.005, and F1 score of 0.984 ± 0.001. Then, we analyzed the ML performance in different regions of the brain and concluded that using the whole brain for depression detection will give the highest accuracy. The proposed method can detect depression among young adults with minimum requirements compared to other related works.

Our proposed work can aid as compliment to the traditional screening tool-based depression diagnosis. This method will be able to help in treatments by cross checking the condition before and after the treatment. Wired EEG headsets are expensive, bulky, and are inconvenient to use but a wireless EEG headset is less expensive and easy to use. Using wireless EEG headset, multiple setups can be arranged which will require less manpower and can automatically screen depression among young adults.

### 4.1. Limitations and Future Work

We have faced a few limitations/challenges during this work. In this work, we have focused on very selective study population for example the subjects are young adults who are private university students from Bangladeshi urban culture belonging to a specific socioeconomical status. So, the study result may be different if the study population belong to different age group or public university going student or has different socioeconomical status. During EEG recording, there were participants with thick or long hair as a result it was difficult for them to put on the headset for better connectivity. Few participants were unable to sit still during EEG recording which created artifacts, so we had to re-record their data. The dataset was imbalanced as the number of control and depressed participants were not equal. We were able to conduct only one session of the EEG recording and because of that we were unable to analyze in different period. So, in the future, we will record the EEG data from the participants at different periods to monitor and analyze the changes. In our next work, we will explore the effects of artifact removal with our dataset to improve the quality of the recorded signal. In the future, we will explore more feature extraction and feature selection methods to improve the performance of ML algorithms. We will also analyze channel selection methods to identify the best combination of channels to improve our findings. We will explore a deep-learning models with our dataset. We will also investigate other mental health issues such as anxiety and stress for screening using EEG and machine learning.

## Figures and Tables

**Figure 3:**
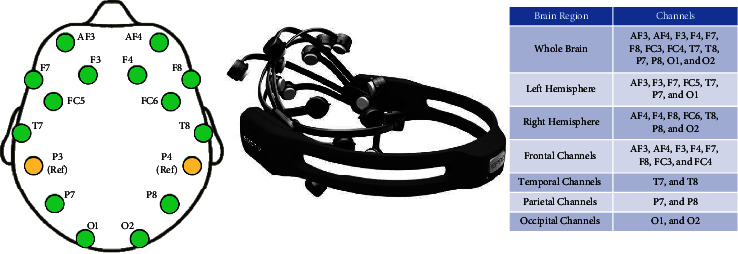
Channel locations, Emotiv headset, and channels at different regions of the brain.

**Table 1:** PHQ9 scores and their corresponding severity levels.

PHQ9 score	Depression level
0–4	No depression
5–9	Mild depression
10–14	Moderate depression
15–19	Moderately severe depression
20–27	Severe depression

**Table 2:** Participant's information about gender, age, PHQ9 score, and group.

Subject ID	Gender	Age (yr.)	PHQ9 score	Group
1	F	24	23	Depressed
2	F	23	21	Depressed
3	F	24	23	Depressed
4	F	23	23	Depressed
5	M	25	24	Depressed
6	F	23	22	Depressed
7	M	20	23	Depressed
8	F	21	25	Depressed
9	F	23	26	Depressed
10	M	21	21	Depressed
11	M	20	23	Depressed
12	F	20	22	Depressed
13	F	22	23	Depressed
14	M	24	22	Depressed
15	F	21	23	Depressed
16	F	19	23	Depressed
17	F	20	21	Depressed
18	F	18	22	Depressed
19	F	20	20	Depressed
20	M	24	5	Control
21	F	22	5	Control
22	M	21	4	Control
23	M	21	2	Control
24	M	25	0	Control
25	M	24	3	Control
26	M	20	3	Control
27	M	22	4	Control
28	M	20	5	Control
29	M	19	4	Control
30	M	21	5	Control
31	F	20	4	Control
32	M	18	5	Control

**Table 3:** Number of samples after segmentation at different lengths.

S/N	Each segment length	Total no. of sample	Total sample no. for depressed group	Total sample no. for control group
1	1 sec (128 samples)	9600	5700	3900
2	2 sec (256 samples)	4800	2850	1950
3	3 sec (384 samples)	3200	1900	1300
4	4 sec (512 samples)	2400	1425	975
5	5 sec (640 samples)	1920	1140	780

**Table 4:** Description of different KNN and SVM classifiers.

KNN			SVM		
S/N	Type	No of neighbors	S/N	Type	Kernel
1	Fine KNN	1	1	Linear SVM	Linear
2	Medium KNN	10	2	Quadratic SVM	Quadratic
3	Coarse KNN	100	3	Cubic SVM	Cubic polynomial
4	Cosine KNN	10	4	Gaussian SVM	Radial basis function
5	Cubic KNN	10			
6	Weighted KNN	10			

**Table 5:** Comparison of classification accuracy with standard deviation of beta band using all classifiers.

Beta band										
Results	Linear SVM	Quadratic SVM	Cubic SVM	Gaussian SVM	Fine KNN	Medium KNN	Coarse KNN	Cosine KNN	Cubic KNN	Weighted KNN
Accuracy	88.02	96.89	97.22	96.96	96.89	96.63	87.39	94.79	95.46	**97.21**
SD	0.24	0.21	0.21	0.17	0.21	0.2	0.26	0.28	0.27	**0.18**

Table 5 shows the average accuracy and the standard deviation results. From there we can observe that the beta band with Cubic SVM 97.22 ± 0.21 accuracy and with weighted KNN gives 97.213 ± 0.18 accuracy. Here, weighted KNN is best as it has a lower standard deviation than Cubic SVM although cubic gives 0.01% higher accuracy. So, the values are bold (having a lower STD represents better robustness of the classifier model) in the table.

**Table 6:** Comparison of classification accuracy with standard deviation of different combinations of sub-bands using all classifiers.

Classifier	Results	ABDT (0.5–30 Hz)	ABT (4–30 Hz)	AB (8–30 Hz)	ABTG (4–64 Hz)	ABG (8–64 Hz)
Linear SVM	Accuracy	79.66	85.14	85.42	85.67	86.76
	SD	0.40	0.30	0.29	0.29	0.21

Quadratic SVM	Accuracy	95.69	97.56	97.86	98.01	98.40
	SD	0.37	0.22	0.16	0.27	0.17

Cubic SVM	Accuracy	95.95	97.67	98.06	97.82	98.39
	SD	0.30	0.23	0.17	0.18	0.22

Gaussian SVM	Accuracy	95.13	97.45	97.96	97.26	97.77
	SD	0.20	0.19	0.14	0.14	0.11

Fine KNN	Accuracy	**96.33**	**98.32**	**98.43**	**98.39**	**98.41**
	SD	**0.25**	**0.13**	**0.15**	**0.14**	**0.17**

Medium KNN	Accuracy	94.67	97.11	97.57	97.35	97.63
	SD	0.22	0.20	0.15	0.14	0.14

Coarse KNN	Accuracy	77.96	76.85	83.38	79.77	81.08
	SD	0.41	0.38	0.33	0.30	0.30

Cosine KNN	Accuracy	94.53	97.09	96.95	97.08	97.15
	SD	0.18	0.23	0.18	0.13	0.13

Cubic KNN	Accuracy	92.91	95.78	96.71	95.93	96.51
	SD	0.28	0.24	0.18	0.20	0.12

Weighted KNN	Accuracy	95.40	97.77	98.23	97.74	98.22
	SD	0.17	0.20	0.21	0.16	0.12

The values in the table are bold because they are the best classifier model overall considering both accuracy and standard deviation.

**Table 7:** Comparison of overall accuracy, precision, NPV, sensitivity, specificity, and F1 score of different combination of sub-bands using fine KNN classifier.

Results		Frequency range			
		ABT (4–30 Hz)	AB (8–30 Hz)	ABTG (4–64 Hz)	ABG (8–64 Hz)
Accuracy	Average	97.94	**98.10**	98.03	98.20
	SD	0.33	**0.11**	0.26	0.30

Precision	Average	0.985	0.984	**0.986**	0.985
	SD	0.004	0.003	**0.003**	0.003

NPV	Average	0.971	**0.977**	0.972	0.978
	SD	0.004	**0.002**	0.007	0.005

Sensitivity	Average	0.980	**0.984**	0.981	0.985
	SD	0.003	**0.002**	0.005	0.004

Specificity	Average	0.978	0.976	**0.979**	0.978
	SD	0.006	0.005	**0.004**	0.004

F1 score	Average	0.983	**0.984**	0.983	0.985
	SD	0.003	**0.001**	0.002	0.003

The best performing values are shown in bold.

**Table 8:** Comparison of overall accuracy, precision, NPV, sensitivity, specificity, and F1 score of different brain regions using fine KNN classifier.

Result		Brain region						
		Whole brain	Left hemisphere	Right hemisphere	Frontal lobe	Temporal lobe	Occipital lobe	Parietal lobe
Accuracy	Average	98.10	96.66	93.66	93.97	83.18	88.03	86.90
	SD	0.11	0.45	0.35	0.40	0.53	0.35	0.51

Precision	Average	0.984	0.973	0.941	0.948	0.857	0.892	0.884
	SD	0.003	0.005	0.005	0.006	0.005	0.003	0.005

NPV	Average	0.977	0.957	0.930	0.928	0.794	0.863	0.847
	SD	0.002	0.007	0.007	0.005	0.009	0.010	0.008

Sensitivity	Average	0.984	0.971	0.953	0.951	0.860	0.909	0.898
	SD	0.002	0.005	0.005	0.004	0.008	0.008	0.006

Specificity	Average	0.976	0.961	0.912	0.924	0.791	0.839	0.827
	SD	0.005	0.007	0.008	0.010	0.009	0.006	0.008

F1 score	Average	0.984	0.972	0.947	0.949	0.859	0.900	0.891
	SD	0.001	0.004	0.003	0.003	0.005	0.003	0.004

**Table 9:** Comparison of this work with other works over the years.

Reference	No. of sub	Age range	Screening tool	EEG recorder	No of channels	Wireless	Classifier	Accuracy (%)
Mumtaz et al. [23]	64	13–77	DSM-IV	Brain master discovery 24e	24 channels	×	LR	90.5
Wu et al. [42]	55	19–40	BDI-II and DSM-IV	HydroCel geodesic sensor net	128 channels	×	SVM	83.64
Bachmann et al. [45]	26	18–66	ICD-10	Neuroscan Synamps2	64 channels	×	LR	92
Peng et al. [48]	55	21–42	PHQ9	HydroCel geodesic sensor net	128 channels	×	SVM	92
Zhu et al. [51]	39	19–23	BDI-II	HydroCel geodesic sensor net	128 channels	×	SVM	83.42
Čukić et al. [53]	41	24–68	ICD-10	Nicolet one	32 channels	×	MLP, LR, DT, and NB	97.56
Liu et al. [55]	39	23–65	HAM-D	Neuroscan quik-cap	64 channels	×	SVM	89.7
Avots et al. [58]	20	24–60	HAM-D	The cadwell easy II	18 channels	×	KNN and DT	95
Nassibi et al. [62]	84	17–20	BDI-II	Neuroscan Synamps2	64 channels	×	NB	91.8
Seal et al. [63]	33	19–36	PHQ9	EEG traveler braintech	32 channels	×	XGBoost	87
**This work**	**32**	**18–25**	**PHQ9**	**Emotiv Epoc+**	**14 channels**	**√**	**Fine KNN**	**98.43**

Bold values represent the current work.

## Data Availability

The recorded EEG data are available from the corresponding author upon request.
